# Hydrogels for Atopic Dermatitis and Wound Management: A Superior Drug Delivery Vehicle

**DOI:** 10.3390/pharmaceutics10020071

**Published:** 2018-06-14

**Authors:** Ian P. Harrison, Fabrizio Spada

**Affiliations:** Department of Research and Development, Ego Pharmaceuticals Pty Ltd., 21-31 Malcolm Road, Braeside, VIC 3195, Australia; ian.harrison@egopharm.com

**Keywords:** hydrogels, skin, wound healing, drug delivery

## Abstract

Wound management, in addition to presenting a significant burden to patients and their families, also contributes significantly to a country’s healthcare costs. Treatment strategies are numerous, but in most cases not ideal. Hydrogels, three-dimensional polymeric materials that can withstand a great degree of swelling without losing structural integrity, are drawing great attention for their use as topical wound management solutions in the form of films and as vehicles for drug delivery, due to their unique properties of high water content, biocompatibility, and flexibility. Hydrogels, both naturally and synthetically derived, can be tuned to respond to specific stimuli such as pH, temperature and light and they are ideally suited as drug delivery vehicles. Here we provide a brief overview of the history and characteristics of hydrogels, assess their uses in wound management and drug delivery, and compare them with other types of common drug delivery vehicle.

## 1. Introduction

The intricate structure of the human skin both repels environmental insults to the barrier and protects the body’s internal organs, two mechanisms crucial to survival. The skin is subjected to an almost constant barrage of potential injuries, from environmental injury due to exposure to the likes of UV radiation, to physical wounds where one or more layers of the skin are cut, broken, or otherwise damaged. Wounds to the skin, if not properly treated, can become infected, further increasing local tissue damage and potentially leading to systemic inflammation and life-threatening immunological responses, such as sepsis in the worst cases [1]. As a result, the process of wound healing is a crucial component of ensuring the host’s continuing health [2].

Wound healing is a rapid, dynamic, and complex process, encompassing multiple, distinct, and overlapping processes, including haemostasis, inflammation, cellular proliferation, and granulation tissue formation and maturation [3]. While members of other species such as fish [3] and amphibians [4] have demonstrated the ability to perfectly regenerate skin, mammals, including humans, experience great difficulty in completely regenerating damaged tissue, especially if the damage is significant. Human wound repair leads to scarring and the loss of skin appendages, such as hair follicles that contribute to normal skin functions (e.g., sensation) [5]. While most cases of wound healing are successful in a basic sense, in that the process completes and the dermal layers are repaired (thus restoring the skins fundamental role of keeping pathogens out and moisture in), some instances of wound healing may become disrupted, leading to chronic wounds, such as pressure ulcers [6], diabetic leg and foot ulcers, and infected wounds [7]. Chronic wounds, or wounds that have not progressed through the ordered healing process [8], exist in a self-perpetuating inflammatory stage [9], prolonging the burden on the patient and their families and on society as a whole, with an estimated AUD$2.85 billion spent annually on chromic wound management in Australia [10]. 

Numerous regimens are employed for treating wounds, from dressings, bandages, and surgery to targeted drug delivery via optimized vehicles (for a recent review on methods employed for the treatment of wounds, see [11]). Of these optimized vehicles, hydrogels in particular are garnering a lot of interest from the medical and pharmaceutical wound care market, because of their unique characteristics of biocompatibility, high water content, and flexibility. This review aims to provide a brief overview of hydrogels, their applications in drug delivery and wound management, and their benefits over other commonly-used drug delivery vehicles.

## 2. A Brief Research History of Hydrogels

**Figure 1:**
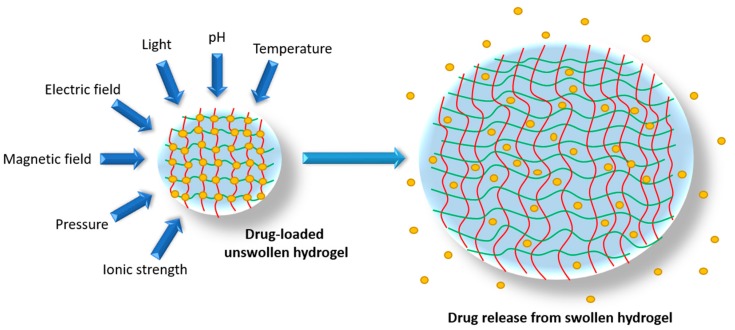
Swelling of a drug delivery hydrogel in response to various chemical and physical stimuli. Red and yellow lines indicate the interwoven matrix structure of a hydrogel, with the yellow dots representing drug molecules.

Hydrogels are hydrophilic, three-dimensional polymeric matrices that are able to absorb and swell with water without dissolving [12,13]. Though a proto-hydrogel concept of a three-dimensional network of hydrophilic natural polymers and gums existed as early as 1894 [14], the first mention of hydrogels that defined them in terms of properties—such as biocompatibility and high water affinity—was in 1960 by Wichterle and Lim [15]. From here on, the focus on hydrogels in research steadily increased until the 1990s. Since then, there has been a near exponential growth in the number of publications on hydrogels [14,16]. This explosion in interest in hydrogels can be ascribed to their evolution over time into the highly versatile products available today. Buwalda and colleagues suggest three distinct phases of hydrogel development [17]. The first phase encompassed the basic concept of Wichterle and Lim, which aimed to develop a relatively simple material with good swelling and mechanical properties. The second stage, beginning in the 1970s, included a more complex type of hydrogel that was able to respond to specific stimuli, such as pH and temperature, and elicit specific responses to these stimuli. The third stage of hydrogel development comprises supramolecular inclusion complexes with excellent biocompatibility and versatility. For example, a complex between Polyethylene Glycol (PEG) and α-cyclodextrins can produce a supramolecular hydrogel that can be tailored to respond to numerous specific stimuli [18], from temperature and pH to electrical fields. This third stage of hydrogel development gave rise to the development of so-called “smart hydrogels”: these are hydrogels with a vast array of tunable properties and possible applications [16], such as drug delivery (Figure 1). 

## 3. Hydrogel Classification

**Figure 2:**
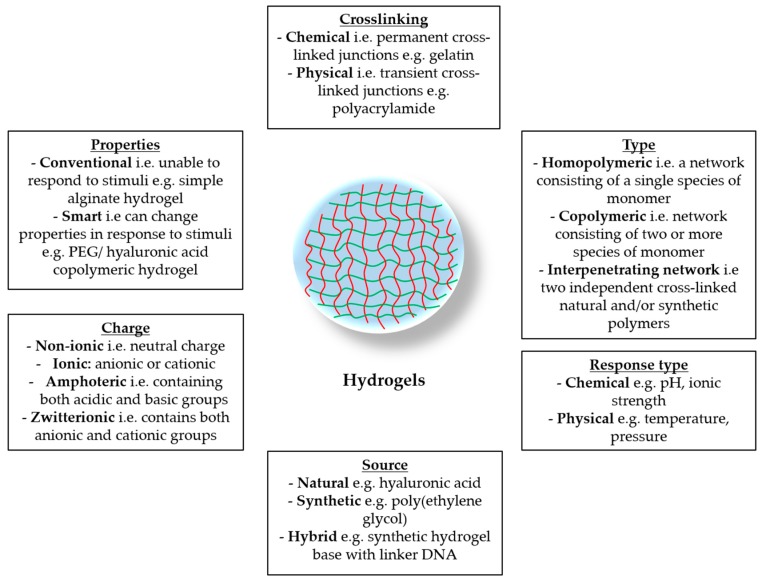
The various parameters by which hydrogels are classified.

Hydrogels can be classified according to various characteristics: their origin (natural, synthetic, or a combination of both), their properties (mechanical or physical), the nature of their polymer side groups (ionic or non-ionic), the type of cross-link (chemical or physical), and their response to various chemical and physical stimuli, to name a few [19] (Figure 2). In the following two sections, we will focus briefly on the categories of hydrogels and their physical and mechanical properties.

Three distinct categories of hydrogels exist: the natural hydrogels that are often composed of polysaccharide chains, such as chitosan [20], cellulose [21] and hyaluronic acid [22], or protein chains such as collagen [23]; the synthetic hydrogels that consist of polymers, such as poly (ethylene glycol) [24] and poly (acrylamide) [25]; and a third group, the hybrid hydrogels, which are composed of a combination of natural and synthetic polymers. Natural and synthetic hydrogels both have their advantages and disadvantages.

### 3.1. Natural Hydrogels

Natural hydrogels offer the greatest biocompatibility, as they are natural components of the Extracellular Matrix (ECM) [16]. Examples include Matrigel™, a basement-membrane extract from Engelbrecht–Holm–Swarm (EHS) mouse sarcoma cells, and gels made from fibrin and hyaluronic acid. Matrigel™ is an oft-used natural hydrogel matrix, and its composition of type IV collagen, laminin, and nidogen make it a very close fit to the in vivo basement membrane [26]. Fibrin gels, made from fibrinogen and thrombin (the key proteins in blood clotting), are well-characterised hydrogels used in the promotion of wound healing [27]. Hydrogels made from hyaluronic acid have numerous applications in the fields of tissue engineering and regenerative medicine through their ability to be tuned by various chemical, mechanical, and spatial cues [22]. Despite the biocompatibility of natural hydrogels, they are limited by the fact that their natural origin means that there will be inherent variability between batches, variables difficult to control between experiments. Additionally, the translational potential of natural hydrogels is limited by the source of the hydrogel to begin with [16,25].

### 3.2. Synthetic Hydrogels

An alternative to natural hydrogels are the synthetic hydrogels, engineered matrices that, by being synthetic, are not susceptible to the limits imposed on natural hydrogels. Examples include Poly (Ethylene Glycol), or PEG, one of the most widely-used synthetic hydrogel materials, owing to its bio-inertness and its effectiveness in suppressing bacterial adhesion, protein adsorption, and cell adhesion [28,29]. Synthetic hydrogels are more reproducible, tend to provide more flexibility for tuning their chemical or mechanical properties [16], and have structures that can be more tightly controlled. The mechanical structure of synthetic hydrogels also tends to be more robust; a hydrogel containing slide-ring polymers for example can stretch to more than ten times its initial length [30]. Given their non-natural origin however, synthetic hydrogels cannot offer the same biocompatibility as that of a natural hydrogel: they will often lack the self-healing abilities of biological tissues, even though they are engineered to mimic them.

### 3.3. Hybrid Hydrogels

The third category of hydrogels, the hybrid hydrogels, use both natural and synthetic polymers to harness the potential of both types [31]. This synergy between the two types of hydrogels can provide the mechanical strength of a synthetic non-natural hydrogel with the biocompatibility and recoverability of natural hydrogels [32]. For example, natural collagen or extracellular matrix-based hydrogels can be strengthened by cross-linking with multi-armed PEG stars containing esters on the termini that react with amine residues on the protein, creating a hybrid hydrogel with a robust synthetic backbone but the same biochemical cues as the natural hydrogel, due to the inertness of PEG [33,34].

## 4. The Properties of Hydrogels

### 4.1. Physical Properties

Given the fact that hydrogels are polymeric matrices swollen with water, the characteristics of the water within a hydrogel will naturally be an important determinant of how a hydrogel functions. When water is first taken in by a dry hydrogel, it is the most polar hydrophilic groups that will interact first with the water molecules and become hydrated, leading to what is termed ‘primary bound water’ [16]. Once these groups are hydrated, the hydrophobic groups are in turn exposed and interact with water molecules, leading to ‘secondary bound water’ [16]. The combination of primary and secondary bound water is known as ‘total bound water’. Osmotic forces will take in additional water, but any additional swelling in the structure will be opposed by the covalent or physical crosslinks so that the swelling level of the hydrogel reaches equilibrium [16]. This excess, or ‘free’, water is assumed to fill the center of larger pores or the space between network chains [16].

### 4.2. Mechanical Properties

The appeal of hydrogels in numerous and varied applications is in large part due to the fact that their mechanical properties can vary considerably depending on requirements. For instance, the rigidity of the structure can be lessened by heating it, while it can be made more rigid by increasing the degree of crosslink within its structure. The pores within a hydrogel structure that are thought to absorb free water when the structure reaches swelling equilibrium can also increase or decrease in size by varying the degree of crosslink within the hydrogel matrix. The structure of conventional hydrogels tends to be more fragile when swollen, but recent studies show that the ability of hydrogels to swell with great amounts of water need not be at the expense of durability [35,36]. It is due to this considerable flexibility in mechanical properties that hydrogels have become a subject of great interest in the fields of wound healing and drug delivery.

## 5. Hydrogels and Wound Healing

As stated previously, wounds, and especially chronic wounds, present a significant burden to the sufferer, their families and the economy. Numerous wound treatment interventions exist in the form of topical pharmacological formulations and wound dressings. The characteristics of the ideal dressing as outlined by Jones and colleagues [37] include the maintenance of high humidity at the wound site, the removal of excess exudate, freedom from particles and toxic contaminants, the ability to be removed without causing further trauma, impermeability to bacteria, comfortability, the allowance of gaseous exchange, and infrequent changes. Though many wound treatment strategies exist, they all invariably lack one or more properties that prevent them from being the optimal strategy. Bandages and dressings, for example, can protect a wound from bacteria, but they need frequent changing and cannot remove excess exudate, maintain a moist environment, nor be removed without causing at least some trauma to the wound. 

Hydrogel-based products, on the other hand, present a more attractive wound management solution. The hydrophilic nature of hydrogels ensures that they are able to retain large amounts of water at the wound site, while their mechanical structure prevents the dissolution of the polymer. The closely woven nature of the hydrogel matrix allows the passage of bioactive molecules, such as antimicrobials and pharmaceutical agents to the wound while preventing bacteria from getting in [38,39]. Additionally, hydrogels do not readily bind to highly hydrophilic surfaces like wounds, so the potential for harm caused by dressing changes is drastically reduced when compared to bandages, gauze, or non-hydrogel films, all of which are either at least low-adherent or not amenable to a constantly moist environment, causing the covering to stick to the wound. Finally, in what is perhaps their most remarkable property, hydrogels can reversibly absorb and release water in response to changes in environmental stimuli such as temperature and light [40].

In a mouse model of diabetic ulcers, Chen et al. found that a biocompatible, multifunctional crosslinker-based temperature-sensitive hydrogel with Bone Marrow-derived Mesenchymal stem Cells (BSMC) inhibited pro-inflammatory M1 macrophage expression at the site and significantly improved wound contraction and healing, compared with control [41]. Histology and immunohistochemistry confirmed that this was due to the BSMC-laden hydrogel, promoting granulation tissue formation, angiogenesis, re-epithelialization, extracellular matrix secretion, and wound contraction. Similarly, Xiao et al. demonstrated that a chitosan-collagen hydrogel with an angiopoietin-1-derived integrin-binding prosurvival peptide significantly accelerated and enhanced wound healing, compared with a clinically-approved collagen wound dressing control in a mouse model of diabetic ulcers [42]. A poly (vinyl alcohol)/chitosan hydrogel containing bee venom has been shown to accelerate healing of diabetic wounds in rats, with an anti-inflammatory effect similar to that of diclofenac gel, the standard nonsteroidal anti-inflammatory drug treatment [43]. Kanokpanont and colleagues reported that a bi-layered wound dressing consisting of a non-adhesive wax-coated silk fibroin fabric layer and a glutaraldehyde-crosslinked silk fibroin/gelatin bioactive layer increased epithelialization and collagen formation and decreased wound size to a greater degree than a clinically-used wound dressing in a model of full-thickness wounds [44]. Seow et al. showed that a cysteine-containing ultrashort peptide hydrogel accelerated re-epithelialization of full-thickness excision wounds in mice compared with controls [45]. In a rat model of wound infection, Zhao et al. found that a thermosensitive hydrogel with a sustained curcumin-releasing profile closed wounds at a quicker rate than gauze and led to improved histological outcomes [46]. In vitro analysis indicated that the hydrogel had distinct antimicrobial, anti-oxidative, and anti-nuclear factor-κB activity. Similarly, Gong and colleagues showed that a curcumin-loaded thermosensitive hydrogel had better tissue adhesiveness than a control dressing and could release curcumin over an extended period [47]. At the wound site, the curcumin-loaded hydrogel group also exhibited greater collagen content, greater wound maturity, better granulation, a decrease in superoxide dismutase, and an increase in catalase. Henderson et al. reported that a sustained delivery of the angiogenic chemokine stromal-derived factor-1 via an alginate hydrogel vehicle significantly decreased the observed wound area on the dorsum of mice and significantly increased endothelial cell invasion into the wound bed, compared with a saline-loaded control [48]. Yasasvini et al. showed that poly (vinyl alcohol) hydrogels loaded with Simvastatin-chitosin microparticles at an optimum low dose significantly improved wound healing in Wistar rats, compared with low-dose ointment and untreated controls [49].

The versatility of hydrogels also make them ideal delivery materials for antibiotics in the treatment of infected wounds. The broad-spectrum antibiotic silver has been used for centuries in the treatment of infections, yet the high reactivity of the silver cation means that its incorporation into delivery vehicles is often quite challenging. Pinto et al. found that a silver-loaded soft agar hydrogel had good antibacterial efficacy at the wound site in a model of skin and soft tissue infections [50], while providing an easier, more stable, and acceptable material to work with. Similarly, a chloramphenicol-loaded 2,3-dialdehyde cellulose hydrogel prepared by Laçin was found to have prolonged antibacterial effects and greater fibroblast adhesion and proliferation than a cellulose control [51]. The author concludes that this hydrogel is ideally suited to wound healing, due to its biodegradability, biocompatibility, and antimicrobial effectiveness.

Hydrogels as synthetic skins in the treatment of wounds have been a subject of study for at least two decades [52]. Kao et al. was able to construct a three-dimensional dermis, using fibroblasts mixed with a biocompatible peptide hydrogel scaffold, which, when combined with keratinocytes, formed a synthetic skin with three to five keratinocyte layers. These layers were found to contain human type 1 collagen, which indicated expression of basement membrane proteins, functional expression around fibroblasts in the dermis, and keratinocyte differentiation in the epidermis [53]. Similarly, Lee et al. found that optimized hydrogel semi-interpenetrating polymer networks of PEG diacrylate and hyaluronic acid were able to support both long-term survival of encapsulated fibroblasts and cell migration [54], results that would have potential in the therapeutic transplantation of cells for wound healing.

The potential of hydrogels in wound healing is not only limited to efficacy: the cost and ease of use of hydrogels as a delivery system can help overcome the limitations of other systems. Murphy et al. found that a hyaluronic acid-based hydrogel containing solubilized amnion membrane not only accelerated wound closure, it also provided an easy-to-use delivery system that overcame the significant cost and handling limitations presented by the placing of thin sheets of living cellularized tissue that had been the preferred treatment strategy [55].

## 6. Hydrogels in Drug Delivery

Hydrogels have become increasingly attractive as vehicles for drug delivery, due to their unique properties. Their highly porous nature allows the loading and releasing of drugs, a property that can be easily tuned by altering the density of cross-links in their matrix structure. Sustained delivery of a drug is a particular advantage offered by hydrogels through the tuning of mechanisms such as diffusion and swelling and by programming responses to environmental stimuli, such as pH or temperature. The versatility of hydrogels also makes them ideal vehicles for proteins and peptides that normally have very short duration of action; conjugation of a drug to PEG for instance can retard kidney filtration and as a result increase plasma half-life of the drug considerably [56]. Previously, hydrogels had been limited to carrying only hydrophilic drugs, due to the limited homogeneity of hydrophobic drugs loaded in hydrogel matrices [57], but recent studies have utilized hydrogels composed of networks of small micelles (around 200 nm) [58] that have a hydrophobic core and hydrophilic shell, allowing the delivery of both hydrophobic and hydrophilic compounds [57,59]. Polo Fonseca and colleagues found in an oral administration simulation that a polyurethane hydrogel was able to deliver the hydrophobic acidic NSAID sodium diclofenac in a sustained fashion for up to 40 h in a neutral solution and to achieve 80% of cumulative release [60]. Pillai et al. developed a folic acid-conjugated cross-linked pH sensitive hydrogel for site-specific delivery of the hydrophobic compound curcumin [61]. This cross-linked conjugated hydrogel showed higher cellular uptake of curcumin than a non-conjugated form. Similarly, Deepa et al. showed in an in vitro study pH-sensitive sustained release of curcumin from a cross-linked hydrogel prepared via inverse emulsion polymerization [62].

A number of studies have examined hydrogel-based products for the transdermal delivery of drugs, a route of administration of obvious importance in the field of wound healing. Carafa et al. found in an in vitro study that a hydrogel composed of two polysaccharides, locust bean gum and xanthan, showed a protective effect on the integrity of drug-loaded niosomes (non-ionic surfactant vesicular structures) for topical application, leading to slower sustained release of the drug-loaded niosomes from the hydrogel system [63]. Transdermal diclofenac transport over 24 h from a solid hydrogel has been shown to be greater than any other known diclofenac formulation [64], with temperature-dependent sustained release of diclofenac, made possible through the entrapment of temperature-responsive nanogels within the solid hydrogel structure. Sun et al. developed composite membranes that cast a linear poly (2-Hydroxyethyl Methacrylate) (pHEMA) solution onto polyester non-woven supports that, depending on the preparation conditions, could be tailored to provide a permeation flux in the range of 4 to 68 µg/cm^2^ per hour of nitroglycerin [65]. Gayet et al. found that high water content (>96%) hydrogels created from a copolymerization of Bovine Serum Albumin (BSA) and PEG allowed the release of soluble and hydrophobic substances from a 2.4 mm-thick hydrogel disk [66]. The authors also showed that the greater the molecular weight of PEG, the more porous the hydrogel. Gabriel et al. showed that a methoxy PEG hexyl substituted poly (lactic acid) composite hydrogel delivered the poorly-solubilised psoriasis drug tacrolimus to the skin of mice with imiquimod-induced psoriasis at a rate twice that of the Protopic™ control, a commercially-available tacrolimus ointment [67]. A hydrogel-thickened microemulsion system for the delivery of the corticosteroid betamethasone diproprionate, which normally has poor permeability through the skin, was found to inhibit inflammation by 72.11% compared with a 43.96% inhibition by a marketed gel in a psoriasis model of rat hind paw edema [68]. Hydrogels as delivery vehicles have also been shown to help improve cosmetic considerations of the skin. Kwankaew et al. reported that a chitosan hydrogel patch incorporating the poorly-solubilized *Artocarpus altilis* heartwood extract (that contains the melanogenesis inhibitor artocarpin) significantly improved hyperpigmentation of the skin via both rapid and slow release of the extract [69].

## 7. Comparing Hydrogels with Other Drug-Delivery Vehicles

The properties of the vehicle used for topical medications can have a significant influence on parameters such as drug delivery, tolerance, and efficacy. In addition, the aesthetic acceptability of these vehicles plays a major role in patient compliance; a vehicle preparation that is difficult to apply or uncomfortable once applied is understandably unappealing to most. Creams and lotions are less greasy than occlusive vehicles and therefore tend to be more appealing to patients, leading to better compliance. They are also easily removed from the skin and allow surface evaporation, which can provide a cooling effect. However, they can cause the formation of mucilaginous slime on the wound surface and require chemical preservatives that may impede wound repair. Surface evaporation also means that they tend to provide less epidermal hydration than occlusive vehicles. Ointments tend to be paraffin-based and form an occlusive barrier over the wound, which can increase both skin hydration and percutaneous drug absorption. Their occlusive, water-free nature protects the skin from aqueous irritants, reduces the risk of sensitization through the lack of preservatives, and provides a longer contact time than creams or lotions. Ointments tend to be greasy and difficult to remove however, which may impact patient compliance, and they lack the ability to provide a cooling effect through surface evaporation, potentially exacerbating discomfort. They also prevent excessive exudate from escaping from a wound, which may cause maceration of healthy skin [70]. 

Hydrogels on the other hand can offer the advantages of creams, lotions and ointments while accounting for their shortcomings (Table 1). In a small split-body, double-blind randomized assessment of the effects of a cream vehicle versus a hydrogel vehicle in 80 men, women, and children with contact dermatitis [71], Draelos found that both investigators and subjects reported that the hydrogel product resulted in a significant improvement in the symptoms of contact dermatitis, compared with the cream-based product. Sabale et al. concluded that a microemulsion-based hydrogel improved the solubility and skin permeability of the broad spectrum antifungal bifonazole, with comparable skin irritancy and antifungal activity to a marketed bifonazole cream [72]. A participant preference study by Trookman et al. reported that the use of a hydrogel formulation containing desonide was found by atopic dermatitis sufferers to be easy to use, comfortable and soothing, disappeared quickly, and was not drying, greasy, or shiny on the skin [73]. The same author reported more recently that desonide hydrogel 0.05% is as effective at reducing the symptoms of mild-to-moderate eczema as a desonide ointment 0.05% preparation, but was rated by patients as significantly better than the ointment for absorbability and lack of greasiness [74]. Similarly, Yentzer et al. found that a hydrogel preparation was consistently rated higher than other vehicles in all categories in a four-week study of desonide treatment for 41 subjects with mild-to-moderate atopic dermatitis [75]. They also found the hydrogel formulation to be efficacious in a shorter timeframe than other vehicles and that patients were more judicious in their adherence to the treatment regimen. The authors conclude that these results may suggest that the reliance on ointments as a first choice in the treatment of atopic dermatitis may actually be counterproductive. A small, single-center, randomized split-body exploratory study of 20 participants with mild-to-moderate atopic dermatitis reported that a hydrogel formulation significantly improved skin hydration at baseline when compared with a moisturizing lotion [76]. The hydrogel also had no significant effect on Transepidermal Water Loss (TEWL), whereas the lotion was found to actually increase TEWL.

We have previously reported that a hydrogel formulation containing 0.1% mometasone furoate is bioequivalent to a 0.1% mometasone furoate lotion, but also provides better moisturisation [77]. Application of the hydrogel resulted in a significant decrease of 43% in TEWL after 2 h, which remained significant (29%) after 24 h. Skin hydration was also significant after 24 h, at 38% above baseline. Based on the similarity of this mometasone furoate hydrogel with a desonide hydrogel, we expected there to be improved patient adherence to the hydrogel application regimen based on previous preference studies with a desonide hydrogel [73,75,78,79]. As desonide is a low-potency topical corticosteroid indicated for use in the treatment of conditions such as atopic dermatitis, it stands to reason that these studies showing patient preference for hydrogels would also apply for similar low-potency topical corticosteroid hydrogel formulations. Recently, we have developed a 1% hydrocortisone hydrogel based on the well-established DermAid™ range. According to the Australian Regulatory Guide for Over-the-Counter Medicines, hydrocortisone is formulation-independent in terms of efficacy and safety, so long as the level of the active ingredient is the same. Comparative diffusion testing using Franz Cell methodology showed that DermAid™ 1% Hydrogel is comparable with other 1% hydrocortisone formulations. As to be expected with different formulations, the results of this Franz Cell testing showed substantial differences in the permeation of hydrocortisone through synthetic membranes of currently registered 1% hydrocortisone products. However, the physiochemical properties that vary the release rates of different hydrocortisone formulations do not necessarily affect bioequivalence or therapeutic equivalence. The stratum corneum has been shown to act as a reservoir and retain topically-applied hydrocortisone [80], and the rate-controlling step is generally the diffusion of the drug from this reservoir, which is relatively slow, rather than the comparatively fast release of the drug from the dosage form. Based on these results, the plethora of efficacy data available on hydrocortisone and the bioequivalence of DermAid™ 1% Hydrogel with other 1% hydrocortisone formulations, DermAid™ 1% Hydrogel offers a more versatile, patient-friendly option for the treatment of mild atopic dermatitis and associated conditions.

## 8. Conclusions

In this review, we provide a brief overview of hydrogels and their applications in wound management and drug delivery for atopic dermatitis. We also briefly outline the pros of hydrogel vehicles compared with the common drug delivery vehicles of creams, lotions, and ointments. Hydrogels show great potential as tools in wound management, as they overcome most of the limitations associated with more traditional forms of wound management solutions like bandages and dressings. Additionally, the biocompatibility, ease-of-use, and incredible versatility and programmability of hydrogels make them ideally suited as vehicles for drug delivery. As alternatives to other drug delivery vehicles, hydrogels have been shown to have at least bioequivalence, and in many cases are more efficacious. They are also consistently rated higher for acceptability by users and may present as the preferred drug delivery vehicle for patient compliance alone. These reasons, and the constant progress being made in hydrogel research, point to hydrogels as the first-choice platform for wound management and drug delivery.

## Figures and Tables

**Table 1:** Advantages and disadvantages of hydrogels compared with the most common drug-delivery vehicles.

Vehicle	Advantages	Disadvantages
Creams and lotions	Not as greasy as occlusive agents, therefore they may have better skin feel and improved patient compliance Water base allows evaporation from the surface of the skin, leading to a cooling effect Easily washed from the skin and clothes	Non-occlusive nature usually leads to less epidermal hydration Non-occlusive nature also means decreased percutaneous drug absorption Water base necessitates the use of preservatives, which may lead to sensitization May cause the formation of mucilaginous slime on the surface of wounds
Ointments	Occlusive base leads to better retention of moisture in the epidermis Water-proof, and thus has a long contact time with the skin Long contact time ensures better percutaneous drug absorption than creams Can protect the skin from aqueous irritants Usually a preservative-free system, thereby reducing the risk of sensitisation	Tend to be very greasy and may have a comparably poor skin feel, which may reduce patient compliance Occlusive nature prevents any cooling effect on the skin Can be difficult to remove from the skin or clothing Oil base tends to prevent exudate from escaping a wound Some oils such as lanolin may lead to sensitisation
Hydrogels	High water content ensures that they are not greasy Better skin feel may improve patient compliance Surface evaporation can lead to a cooling effect on the skin Improves skin hydration and reduces transepidermal water loss Improved drug absorption as contact time tends to be longer than creams or lotions Easily removed from the skin or clothing Natural hydrogels tend to be extremely biocompatible Synthetic hydrogels are hugely tunable, with the ability to respond to many stimuli Tunable drug delivery capabilities mean that drugs can be delivered to the area when needed	Conventional hydrogels tend to be fragile Can be expensive, especially tunable smart hydrogels Synthetic hydrogels are not as biocompatible as natural hydrogels
